# CircRNA DONSON contributes to cisplatin resistance in gastric cancer cells by regulating miR-802/BMI1 axis

**DOI:** 10.1186/s12935-020-01358-w

**Published:** 2020-06-22

**Authors:** Yong Liu, Jianzhong Xu, Min Jiang, Lingna Ni, Yang Ling

**Affiliations:** Department of Oncology, The Third Affiliated Hospital of Soochow University (Changzhou Tumor Hospital Affiliated to Soochow University), No. 68, Honghe Road, Changzhou, 213000 Jiangsu China

**Keywords:** circDONSON, miR-802, BMI1, Gastric cancer, Cisplatin resistance

## Abstract

**Background:**

Circular RNA downstream neighbor of SON (circDONSON) has been revealed to promote gastric cancer (GC) growth and invasion, while the role and molecular mechanism underlying circDONSON in GC cisplatin (DDP) resistance remain unclear.

**Methods:**

Levels of circDONSON, microRNA (miR)-802, and B lymphoma Mo-MLV insertion region 1 (BMI1) mRNA were detected using quantitative real-time polymerase chain reaction. Cell viability and apoptosis were measured by cell counting kit-8 assay, colony formation assay and flow cytometry, respectively. Protein levels of BMI1, Cyclin D1, p27, Caspase-3 Cleavage and Caspase-9 Cleavage were determined by western blot. The interaction between miR-802 and circDONSON or BMI1 was confirmed by dual-luciferase reporter assay. In vivo experiments were conducted via the murine xenograft model.

**Results:**

CircDONSON was elevated in GC tissues and cell lines, especially in DDP-resistant GC tissues and cells. Knockdown of circDONSON sensitized GC cells to DDP by inhibiting cell viability and promoting cell apoptosis in vitro. Further mechanism-related investigations suggested that circDONSON functioned as “sponge” by competing for miR-802 binding to modulate its target BMI1. Silencing miR-802 reversed the inhibition of DDP-resistance in GC cells induced by circDONSON down-regulation. Besides, miR-802 alleviated DDP resistance in GC cells by targeting BMI1. Functionally, circDONSON knockdown enhanced the cytotoxicity of DDP in GC in vivo.

**Conclusion:**

Our findings demonstrated circDONSON promoted cisplatin resistance in gastric cancer cells by regulating miR-802/BMI1 axis, shedding light on the development of a novel therapeutic strategy to overcome chemoresistance in gastric cancer patients.

## Background

Gastric cancer (GC) is the third-most common cause of cancer-related death worldwide, and there are more than 1,000,000 cases with gastric cancer in 2018, nearly two-thirds of which occurred in developing countries [1]. Despite improvements in the overall survival of gastric cancer patients using preoperative and postoperative combined chemotherapy, the prognosis of gastric cancer patients is still poor, mainly due to drug resistance and tumor metastasis, which are major obstacles for effective cancer chemotherapy [2, 3]. Cisplatin (DDP) is a mainly used chemotherapy drug in the treatment of gastric cancer patients, especially in advanced gastric cancer patients [4]. However, DDP resistance gradually emerged in gastric cancer patients, which led to a low inhibitory rate, eventfully chemotherapy failure [5]. Thus, it is of great significance to better understand the underlying mechanisms of DDP resistance in gastric cancer.

Circular RNAs (circRNAs) are a new type of non-protein coding RNAs with the covalently closed loop structures [6]. It has been revealed that circRNAs can modulate a series of biological processes, including cell proliferation, migration, invasion, immune responses and oxidative stress [7, 8]. Besides that, recent studies also indicated circRNAs participate in chemoresistance in human cancers. For example, circPVT1 was up-regulated in osteosarcoma, and enhanced the resistance of osteosarcoma cells to DDP via up-regulating the expression of ABCB1 [9]. Knockdown of circEIF6 was found to weaken the resistance of human thyroid carcinoma cells to DDP through sponging miR-144-3p [10]. Thus, circRNAs are ideal candidates for future chemoresistance interventions. Circular RNA downstream neighbor of SON (circDONSON) is novel identified circRNA, which is derived from back-splicing of DONSON mRNA. Recently, Ding et al. demonstrated that circDONSON expression was elevated in gastric cancer, and high circDONSON expression was closely associated with unfavorable prognosis; importantly, knockdown of circDONSON significantly inhibited tumor growth in vivo and suppressed malignant biological behaviors of gastric cancer cells in vitro [11]. Thus, circDONSON may be an important regulator in gastric cancer progression. However, whether circDONSON serves as a useful circRNA for intervening DDP resistance in gastric cancer has not been well investigated.

Herein, this study aimed to explore the expression patterns of circDONSON in DDP-resistance tissues and cells, investigated the function as well as related target genes and molecular mechanism underlying circDONSON in DDP resistance in gastric cancer.

## Materials and methods

### Clinical samples

A total of 60 cases of normal gastric mucosa from noncancerous patients and gastric cancer tissues were obtained from The Third Affiliated Hospital of Soochow University and immediately stored at − 80 °C until RNA extraction. The clinical characteristics of those patients were presented in Table 1. All enrolled patients have only underwent DDP-based chemotherapy before surgery, and then were divided into two groups: drug sensitive group (N = 35, Treatment-responsive) and drug resistance group (N = 25, Treatment-resistant) depending on the sensitivity of gastric cancer patients to DDP. This study was permitted by the Ethics Committee of The Third Affiliated Hospital of Soochow University and all patients had provided written informed consent.

**Table 1:** Clinical characteristics in 60 cases of gastric cancer samples

Clinicopathological factors	Number
Age (years)	
< 50	32
≥ 50	28
Gender	
Female	26
Male	34
Tumor size (cm)	
> 3	42
≤ 3	18
Lymph node metastasis	
Negative	39
Positive	21
TNM stage	
I–II	26
III–IV	34

### Cell culture

The human gastric epithelial immortalized cell lines GES-1 and human gastric cancer cell lines (AGS and HGC-27) was obtained from Beijing Institute for Cancer Research Collection, and then maintained in RPMI-1640 Medium (Invitrogen, Carlsbad, CA, USA) containing 10% fetal bovine serum (FBS), and 1% penicillin/streptomycin in culture flasks with 5% CO_2_ at 37 °C.

To generate DDP-resistance (AGS/DDP and HGC-27/DDP) gastric cancer cells, parental AGS and HGC-27 cell lines at the logarithmic growth phase were digested with trypsin, and exposed to increasing concentrations of DDP (0.5–5 µM) (Sigma, San Francisco, CA, USA) over several months. DDP-resistance gastric cancer cells were routinely cultured in the same media added with 1 µM DDP. Resistant cells were further cultured in drug-free RPMI-1640 containing 5% FBS for 1 week before follow-up experiments.

### Quantitative real-time polymerase chain reaction (qRT-PCR)

The extraction of total RNA from tissues and cells was conducted with the help of TRIzol reagent (Invitrogen) according to the standard procedure. The reverse transcription reactions were reversely transcribed from RNA using the Reverse Transcription System Kit (Takara, Dalian, China), then cDNA amplification was performed using SYBR Green I (Takara) on the ABI7500 system. The relative expression was calculated by the 2^−ΔΔCt^ method with glyceraldehyde-3-phosphate dehydrogenase (GAPDH) or U6 small nuclear B noncoding RNA (U6) as a normalization control. Primers were presented as followed: circDONSON: F 5′-ATATCAGGCTCGGAGAATGATACATGTGAAGT-3′, R 5′-CTTAAGCATTTGCATTGTGGCACCTCGG-3′; miR-802: F 5′-TCCAGTGCCAGAAATGAACC-3′, R 5′-CTGAACCACAGTTACAGAGC-3′. B lymphoma Mo-MLV insertion region 1 (BMI1), F 5′-TGGCTCGCATTCATTTTCTG-3′, and R 5′-AGTAGTGGTCTGGTCTTGTG-3′; U6: F 5′-CTCGCTTCGGCAGCACA-3′, R 5′-CGCTTCACGAATTTGCGTGTCAT-3′; GADPH: F 5′-CCCACATGGCCTCCAAGGAGTA-3′, R 5′-GTGTACATGGCAACTGTGAGGAGG-3′.

### Cell transfection

The miR-802 mimic, miR-802 inhibitor (anti-miR-802) and their negative control (miR-NC mimic, anti-miR-NC) were achieved by RiboBio (Guangzhou, China). Small interfering RNA (siRNA) sequences targeting circDONSON (si-circDONSON, 5′-AUGUAAAGGUGUUGAUUCCUU-3′), siRNA negative control (si-NC, 5′-UUCUCCGAACGUGUCACGU-3′), pcDNA-BMI1 overexpression vector (pcDNA-BMI1), empty vector (pcDNA-NC), and lentiviral encoding either short hairpin RNA (shRNA)-targeting circDONSON (sh-circDONSON) or a scrambled control sequence (sh-NC) were designed and synthesized by Invitrogen (Carlsbad, CA, USA). Then transfection of these mimics or vectors was carried out using Lipofectamine 2000 (Invitrogen).

### Cell viability assay

Following transfection for 48 h, resistant cells were placed on a 96-well plate (5000 cell/well) overnight, and then incubated with increasing concentrations of DDP (0.125, 0.25, 0.5, 1, 2, 4, or 8 µM) for another 48 h, respectively. Subsequently, per well was added with 10 μL counting kit-8 (CCK-8) solution (Beyotime, Shanghai, China) and interacted for about 2 h. Finally, the optical density at 450 nm was examined by a microplate reader. Besides that, the half maximal inhibitory concentration (IC_50_) value of DDP was calculated according to the relative survival curve.

### Colony formation assay

After transfection, resistant cells (800/well) were seed into a 6-well plate containing with 1 µM DDP and maintained for 21 days. Then cells were fixed with methanol and stained with 0.1% crystal violet (Sigma). Finally, the visible colonies (≥ 50 cells) were counted and the typical images were photographed.

### Flow cytometry

Transfected resistant cells (1 × 10^6^ cells/mL) were seeded into 6-well plates overnight and treated with 1 µM DDP for 48 h. Then cells collected and resuspended in 400 μL binding buffer, and double-stained with 5 μL Annexin V-FITC and 5 μL propidium iodide (PI) (BD Biosciences, Shanghai, China). Finally, apoptotic rate was measured using a flow cytometry.

### Western blot

Proteins were isolated using RIPA lysis buffer (Beyotime). After the qualification of protein concentration with the bicinchoninic acid assay, approximately 30 μg of extracted protein was added on Sodium dodecyl sulfate–polyacrylamide gel electrophoresis (SDS-PAGE) for separating and then transferred to polyvinylidene difluoride (PVDF) membranes. Subsequently, the membranes were blocked with 5% skim milk powder for 1 h at room temperature and then incubated with primary antibody against Cyclin D1 (1:20000, ab134175, Abcam, Cambridge, MA, USA), p27 (1:5000, ab32034, Abcam), BMI1 (1:3000, ab14389, Abcam), Caspase-3 Cleavage (Cas-3 Cleavage) (1:1000, Cat#:9664, Cell Signaling, Danvers, MA, USA), Caspase-9 Cleavage (Cas-9 Cleavage) (1:1000, Cat#:9505, Cell Signaling) and GAPDH (1:10000, ab181602, Abcam), followed by incubation with HRP-conjugated antibody (1:1000, ab9482, Abcam). Finally, blot bands were visualized and quantitated using the electrochemiluminescence method. GAPDH, came from the same gel as the target protein, was used as a normalization control.

### Dual-luciferase reporter assay

Putative wild-type (WT) and mutant (MUT) miR-802-binding sites in the circDONSON mRNA or BMI1 3′UTR, termed WT-circDONSON or MUT-circDONSON and WT-BMI1 or MUT-BMI1, were cloned into a pmirGLO-report luciferase vector (Promega, Madison, WI, USA). Then cells were cultured in 24-well plates and co-transfected with these reporter plasmids in the presence of miR-802 mimics or miR-NC mimic using Lipofectamine™ 2000 (Invitrogen). The luciferase activity was detected after 48 h transfection with a dual luciferase assay kit (Promega).

### In vivo chemosensitivity assay

The study was approved by the Animal Care and Ethics Committee of The Third Affiliated Hospital of Soochow University, and manipulated in line with the National Institutes of Health animal use guidelines. BALB/c nude mice (4-weeks-old, N = 12) were divided into four groups of three mice each. HGC-27/DDP cells stably infected with sh-circDONSON or sh-NC were subcutaneously injected into right-side flanks of each mouse to establish xenografts. When the tumor volume reached 50 mm^3^, DDP was intraperitoneally injected into mice at a dose of 3 mg/kg every 4 days according to the indicated groups: sh-NC + PBS, sh-NC + DDP, sh-circDONSON + PBS, sh-circDONSON + DDP. The volume of tumors was calculated every 7 days. At day 28, mice were killed and tumor masses were dissected, weighed and harvested for subsequent molecular analyses.

### Statistical analysis

Data form three independent experiments was expressed as the mean ± standard deviation (SD). Group comparison was conducted on GraphPad Prism 7 software using Student’s *t*-test or one-way analysis of variance (ANOVA) followed by Tukey’s test. Correlation analyses were conducted by Spearman rank correlation. *P* values < 0.05 was considered as statistically significant.

## Results

### CircDONSON is elevated in DDP-resistant GC tissues and cell lines

**Fig. 1:**
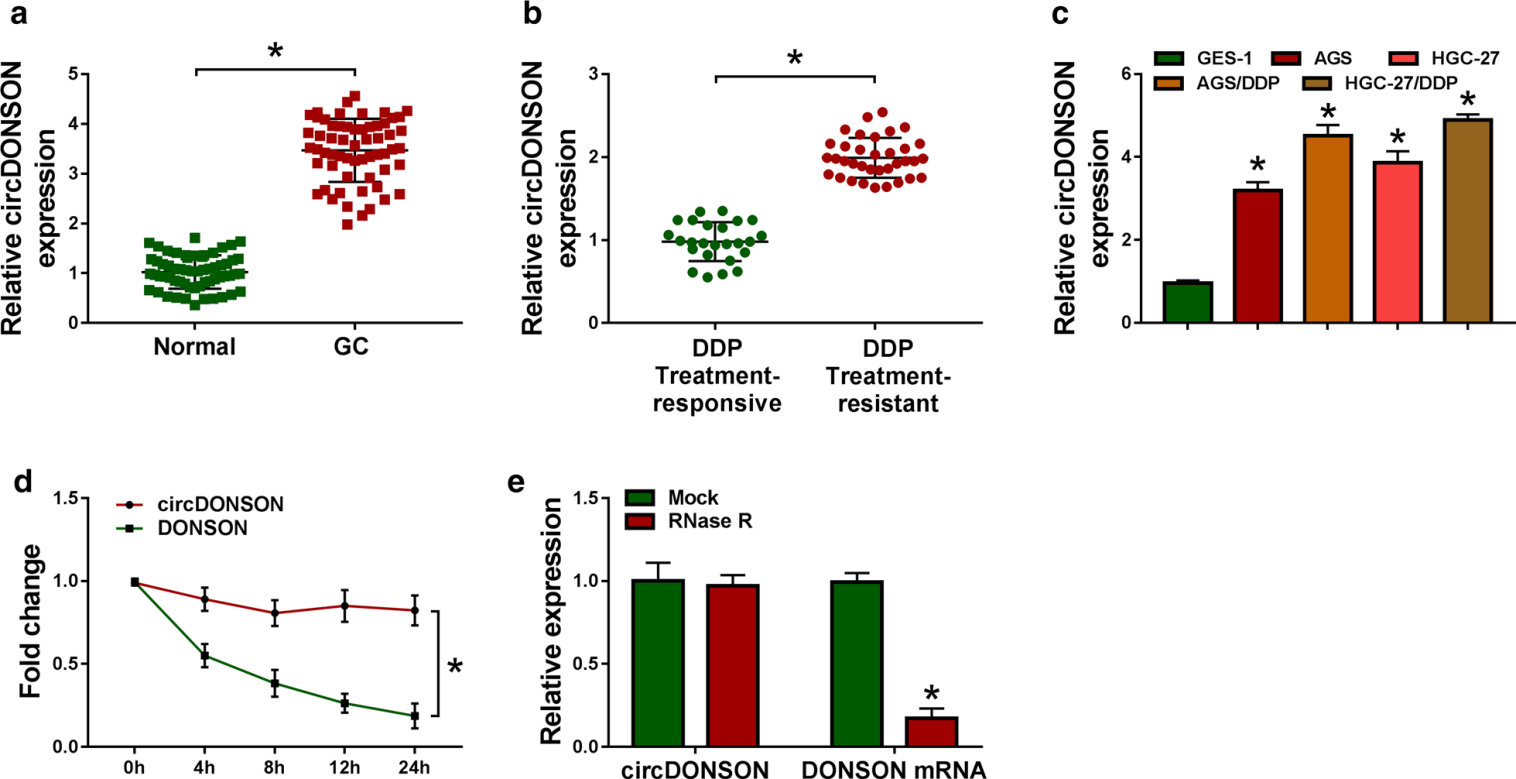
CircDONSON is elevated in DDP-resistant GC tissues and cell lines. **a**, **b** qRT-PCR analysis of circDONSON expression in normal gastric mucosa from noncancerous patients and gastric cancer tissues (N = 60), as well as in DDP responsive (N = 35) and non-responsive GC tissues (N = 35). **c** qRT-PCR analysis of circDONSON expression in gastric epithelial immortalized cell lines GES-1, GC cell lines (AGS and HGC-27), and DDP-resistant GC cell lines (AGS/DDP and HGC-27/DDP). **d** qRT-PCR analysis of circDONSON and linear DONSON expression in AGS cells treated with actinomycin D (2 μg/mL). **e** qRT-PCR analysis of circDONSON and linear DONSON expression after treatment with RNase R (10U/3 μg) in AGS cells. n = 3, *P < 0.05

To investigate the impact of circDONSON on DDP resistance in GC, the level of circDONSON was firstly detected. As shown by qRT-PCR analysis, circDONSON expression was significantly elevated in GC tissues, in particular, in DDP-resistant GC tissues (Fig. 1a, b). Similarly, it was also found circDONSON was increased in GC cell lines (AGS and HGC-27) relative to gastric epithelial immortalized cell lines GES-1; moreover, by contrast with parental AGS and HGC-27 cells, circDONSON expression was higher in DDP-resistant GC cells (AGS/DDP and HGC-27/DDP) (Fig. 1c). Thus, circDONSON increase might be associated with DDP resistance in GC. Subsequently, the stability and localization of circDONSON were investigated. We found the half-life of circDONSON exceeded 24 h, while that of linear DONSON showed only about 4 h after treatment with Actinomycin D treatment in AGS cells (Fig. 1d), implying the high stability of circDONSON. Meanwhile, total RNA from proliferating AGS cells was treated with RNase R, and qRT-PCR analysis showed circDONSON resisted to the degradation induced by RNase R (Fig. 1e), suggesting circDONSON stably functioned as a typical circRNA.

### CircDONSON knockdown inhibits DDP resistance of GC cells in vitro

**Fig. 2:**
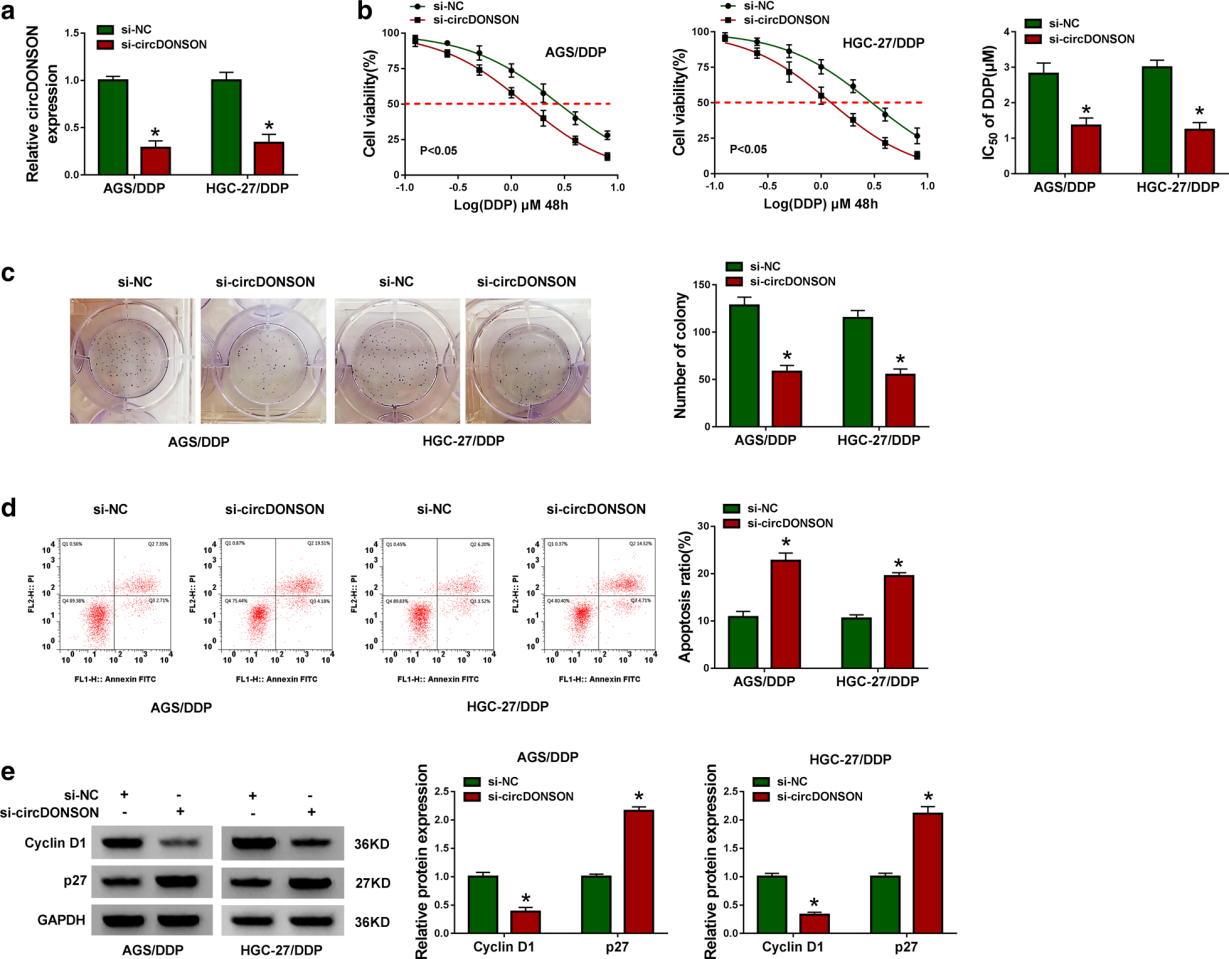
CircDONSON knockdown inhibits DDP resistance of GC cells in vitro. AGS/DDP and HGC-27/DDP cells were transfected with si-NC or si-circDONSON. After transfection, **a** qRT-PCR analysis of circDONSON expression in AGS/DDP and HGC-27/DDP cells; **b** CCK-8 assay of the viability and IC_50_ value of AGS/DDP and HGC-27/DDP cells after exposure to a series dose of DDP (0.125, 0.25, 0.5, 1, 2, 4, or 8 µM); **c** colony formation assay of the colony-forming ability of AGS/DDP and HGC-27/DDP cells under 1 µM DDP treatment; **d** flow cytometry of the apoptosis of AGS/DDP and HGC-27/DDP cells under 1 µM DDP treatment; **e** western blot analysis of Cyclin D1 and p27 levels in AGS/DDP and HGC-27/DDP cells. n = 3, *P < 0.05

It had been proved that circDONSON was elevated in DDP-resistant GC tissues and cells, thus, further cellular experiments were carried out to investigate the action of circDONSON on DDP resistance in GC cells. Si-circDONSON or si-NC was used to knock down circDONSON in DDP-resistant GC cells, as expected, circDONSON level was significantly decreased in AGS/DDP and HGC-27/DDP cells when transfected with si-circDONSON (Fig. 2a). Afterwards, CCK-8 assay exhibited that circDONSON knockdown combined with increasing doses of DDP (0.125, 0.25, 0.5, 1, 2, 4, or 8 µM) gradually inhibited the viability of AGS/DDP and HGC-27/DDP cells, besides, the IC_50_ value of DDP was decreased in si-circDONSON group compared to si-NC group (Fig. 2b). Also, colony formation assay showed circDONSON knockdown combined with 1 µM DDP treatment reduced the number of colonies formed (Fig. 2c). Meanwhile, the apoptosis rate of AGS/DDP and HGC-27/DDP cells was increased under si-circDONSON combined with 1 µM DDP treatment (Fig. 2d), and the percentages and quantification for all 4 quadrants were presented in Additional file 1. Moreover, we also proved that knockdown of circDONSON up-regulated the expression levels of Caspase-3 Cleavage, Caspase-9 Cleavage (Additional file 2: Fig. S1) and p27, but decreased Cyclin D1 expression compared with that of control group in AGS/DDP and HGC-27/DDP cells (Fig. 2e), further indicating the effects of si-circDONSON on the phenotype changes of AGS/DDP and HGC-27/DDP cells. What’s more, through using another siRNA targeting circDONSON, we also proved that circDONSON down-regulation reduced IC50 of cells to DDP, suppressed cell viability and promoted cell apoptosis (Additional file 3: Fig. S2). Taken together, knockdown of circDONSON restored the sensitivity of DDP-resistant cells to DDP.

### MiR-802 is a target of circDONSON in GC cells

**Fig. 3:**
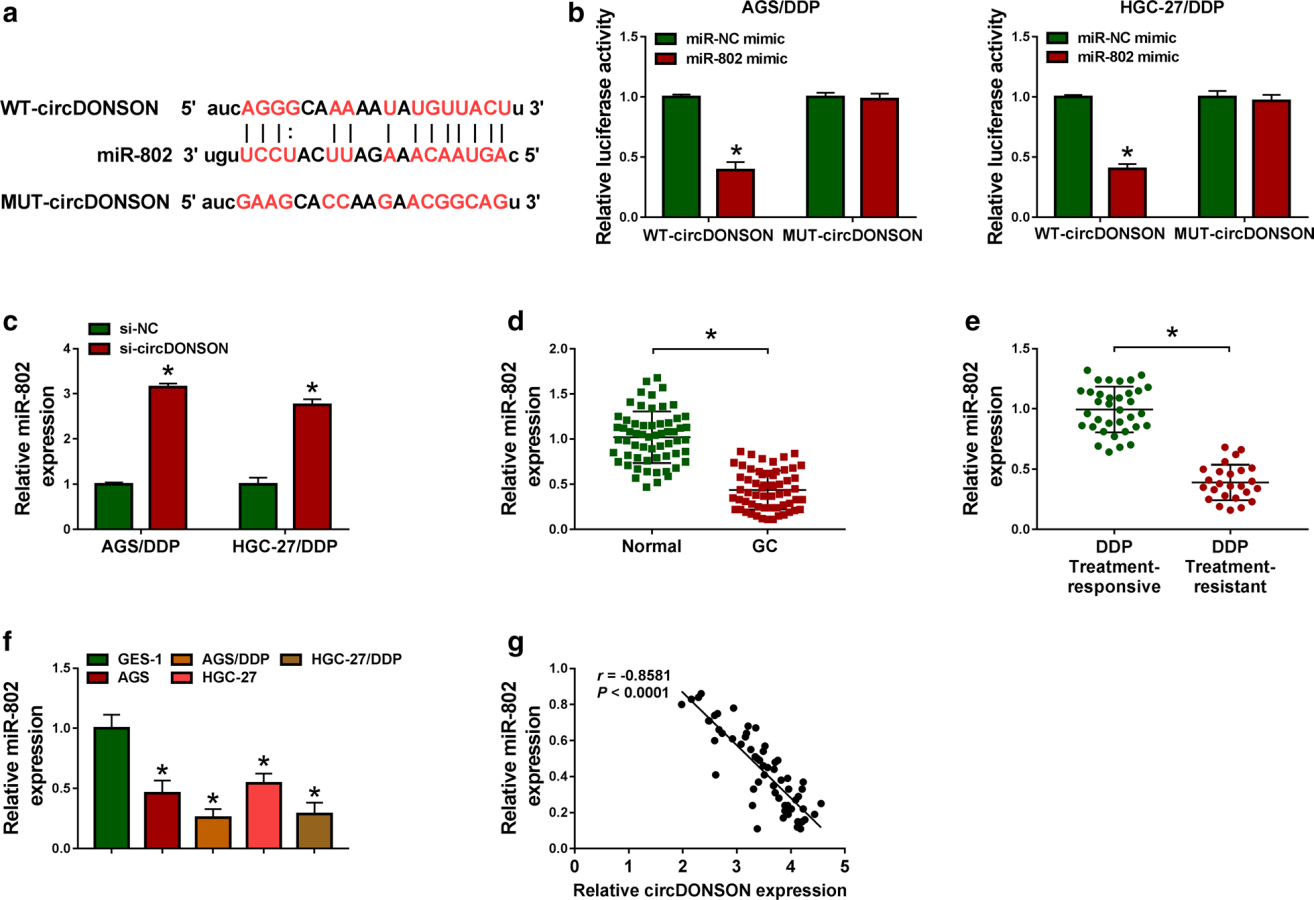
MiR-802 is a target of circDONSON in GC cells. **a** Sequence alignment of miR-802 with the putative binding sites in circDONSON. **b** Dual-luciferase reporter assay in AGS/DDP and HGC-27/DDP cells co-transfected with the reporter plasmid (or the corresponding mutant reporter) and miR-802 mimic or miR-NC mimic. **c** qRT-PCR analysis of miR-802 in AGS/DDP and HGC-27/DDP cells transfected with si-NC or si-circDONSON. **d**, **e** qRT-PCR analysis of miR-802 expression in normal gastric mucosa from noncancerous patients and gastric cancer tissues (N = 60), as well as in DDP responsive (N = 35) and non-responsive GC tissues (N = 25). **f** qRT-PCR analysis of miR-802 expression in normal GES-1 cells, GC cell lines (AGS and HGC-27), and DDP-resistant GC cell lines (AGS/DDP and HGC-27/DDP). **g** Correlation analysis between miR-802 and circDONSON expression in GC tissues. n = 3, *P < 0.05

The molecular mechanism underlying the action of circDONSON on DDP resistance in GC cells was investigated. According to the prediction of StarBase database, the potential binding sits of miR-802 in circDONSON were observed (Fig. 3a). Immediately, a significant reduction of luciferase activity in AGS/DDP and HGC-27/DDP cells co-transfected with WT-circDONSON and miR-802 confirmed the direct interaction between circDONSON and miR-802 (Fig. 3b). Besides, it was also proved that circDONSON knockdown up-regulated miR-802 expression in DDP-resistant cells (Fig. 3c, nearly threefolds). MiR-802 was found to be down-regulated in GC tissues, especially in DDP-resistant GC tissues (Fig. 3d, e). Similarly, it was also decreased in AGS/DDP and HGC-27/DDP cells relative to their parental GC cells (Fig. 3f). In addition, a negative correlation between circDONSON and miR-802 expression in GC tissues was observed (Fig. 3g). Thus, we confirmed that circDONSON directly bound to miR-802 and negatively regulated its expression.

### Silencing circDONSON sensitizes GC cells to DDP by targeting miR-802

**Fig. 4:**
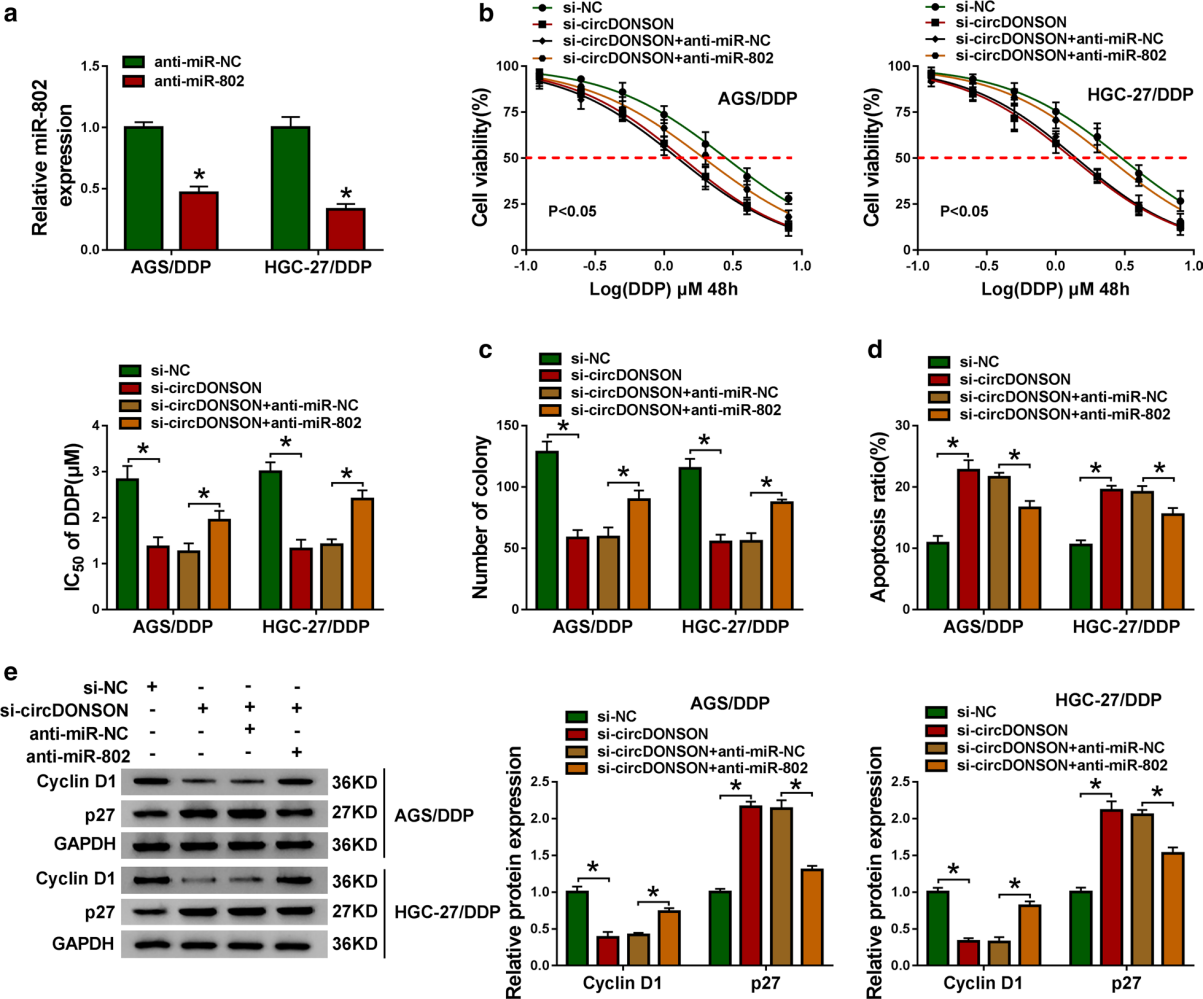
Silencing circDONSON sensitizes GC cells to DDP by targeting miR-802. **a** qRT-PCR analysis of miR-802 in AGS/DDP and HGC-27/DDP cells transfected with anti-miR-802 or anti-miR-NC. AGS/DDP and HGC-27/DDP cells were transfected with si-NC, si-circDONSON, si-circDONSON + anti-miR-NC, or si-circDONSON + anti-miR-802. **b** CCK-8 assay of cell proliferation capacity and IC_50_ value of DDP in AGS/DDP and HGC-27/DDP cells after treatment with a series dose of DDP (0.125, 0.25, 0.5, 1, 2, 4, or 8 µM). **c** Colony formation assay of the colony-forming ability of AGS/DDP and HGC-27/DDP cells under 1 µM DDP treatment. **d** Apoptosis analysis of AGS/DDP and HGC-27/DDP cells under 1 µM DDP treatment using flow cytometry. **e** Levels detection of Cyclin D1 and p27 protein in AGS/DDP and HGC-27/DDP cells with western blot. n = 3, *P < 0.05

Given that circDONSON directly targeted miR-802, whether miR-802 involved in the action of circDONSON on DDP resistance of GC cells was explored. First, we found anti-miR-802 induced significant reduction of miR-802 levels in AGS/DDP and HGC-27/DDP cells compared with anti-miR-NC (Fig. 4a). Then AGS/DDP and HGC-27/DDP cells were transfected with si-NC, si-circDONSON, si-circDONSON + anti-miR-NC, or si-circDONSON + anti-miR-802 to conduct rescue assay. In the CCK-8 and colony formation assays, si-circDONSON decreased the IC_50_ value of DDP and cell proliferation, which were notably reversed by miR-802 inhibition in AGS/DDP and HGC-27/DDP cells (Fig. 4b, c). The data from flow cytometry suggested that si-circDONSON-elicited apoptosis was evidently decreased after the introduction of anti-miR-802 in AGS/DDP and HGC-27/DDP cells (Fig. 4d). Western blot analysis also showed silencing miR-802 attenuated si-circDONSON-induced decrease of Cyclin D1 level and increase of p27 level in DDP-resistant cells (Fig. 4e). Collectively, these findings demonstrated that miR-802 mediated the effects of circDONSON on the DDP resistance of GC cells.

### BMI1 is a target of miR-802 in GC cells

**Fig. 5:**
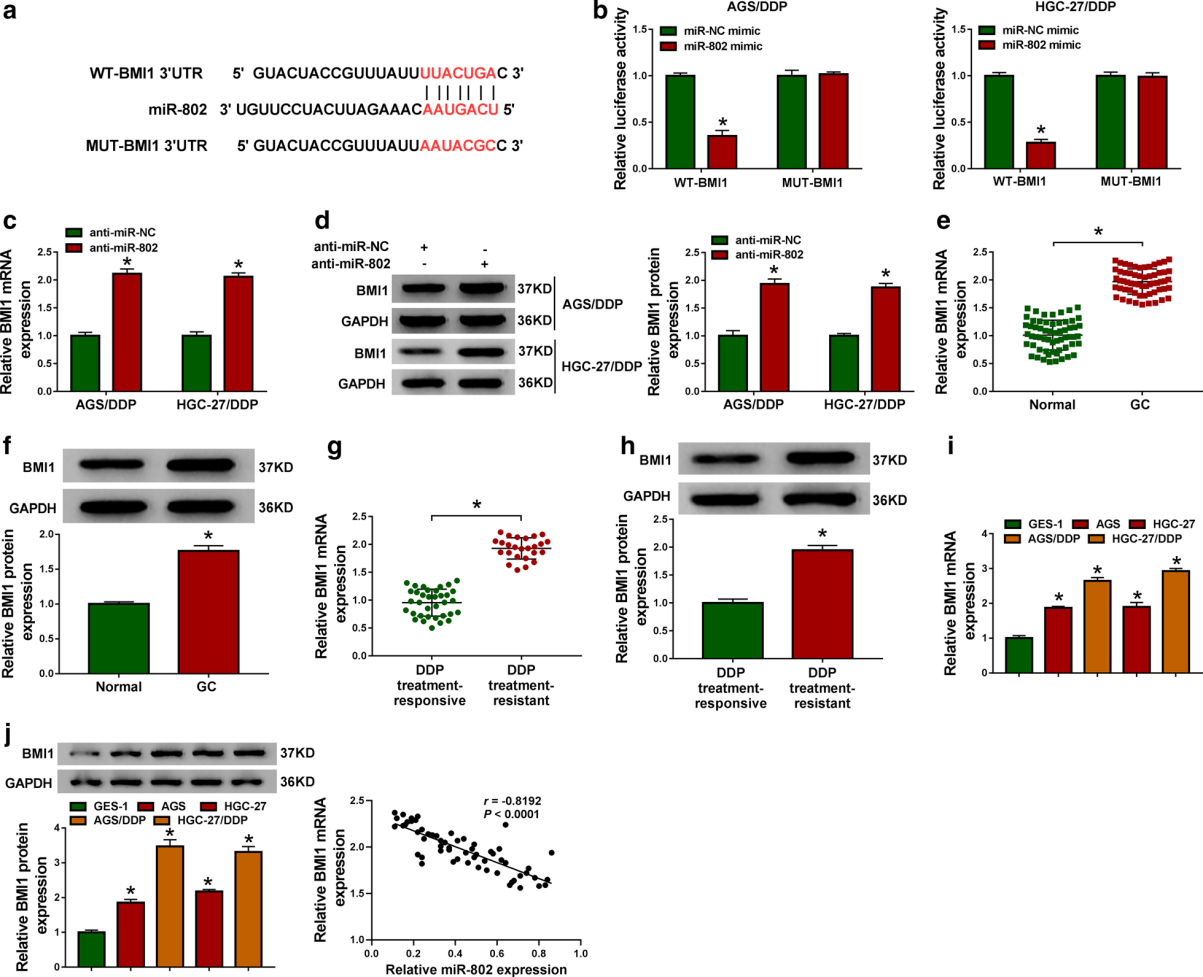
BMI1 is a target of miR-802 in GC cells. **a** Schematic of the binding sites between miR-802 and BMI1. **b** Dual-luciferase reporter assay in AGS/DDP and HGC-27/DDP cells co-transfected with the reporter plasmid and indicated miRNAs. **c**, **d** Levels detection of BMI1 mRNA and protein in AGS/DDP and HGC-27/DDP cells transfected with anti-miR-802 or anti-miR-NC using qRT-PCR and western blot. **e**–**h** Measurement of the mRNA and protein levels of BMI1 in normal gastric mucosa from noncancerous patients and gastric cancer tissues (N = 60), as well as in DDP responsive (N = 35) and non-responsive GC tissues (N = 25) using qRT-PCR and western blot. **i**, **j** Levels detection of the mRNA and protein of BMI1 in normal GES-1 cells, GC cell lines (AGS and HGC-27), and DDP-resistant GC cell lines (AGS/DDP and HGC-27/DDP) using qRT-PCR and western blot. **k** Correlation analysis between miR-802 and BMI1 expression in GC tissues. n = 3, *P < 0.05

Next, the downstream genes of miR-802 in GC cells was investigated through searching TargetScan database, and BMI1 was identified as a potential target of miR-802 (Fig. 5a). Later, a dual luciferase reporter assay was performed and results indicated miR-802 negatively modulated the luciferase activity of WT-BMI1 rather than that of MUT-BMI1 in AGS/DDP and HGC-27/DDP cells (Fig. 5b). Moreover, BMI1 was up-regulated by miR-802 inhibition (Fig. 5c, d). After that, we discovered that BMI1 was elevated in GC tissues (Fig. 5e, f), especially in non-responders relative to responders (Fig. 5g, h), meanwhile, BMI1 expression was increased more in AGS/DDP and HGC-27/DDP cells than that in the AGS and HGC-27 control group (Fig. 5i, j). Additionally, it was also proved that miR-802 expression was negatively correlated with BMI1 expression in GC tissues (Fig. 5k). Therefore, we verified that miR-802 directly targeted BMI1 and inhibited its expression in GC cells.

### MiR-802 alleviates DDP resistance in GC cells by targeting BMI1

**Fig. 6:**
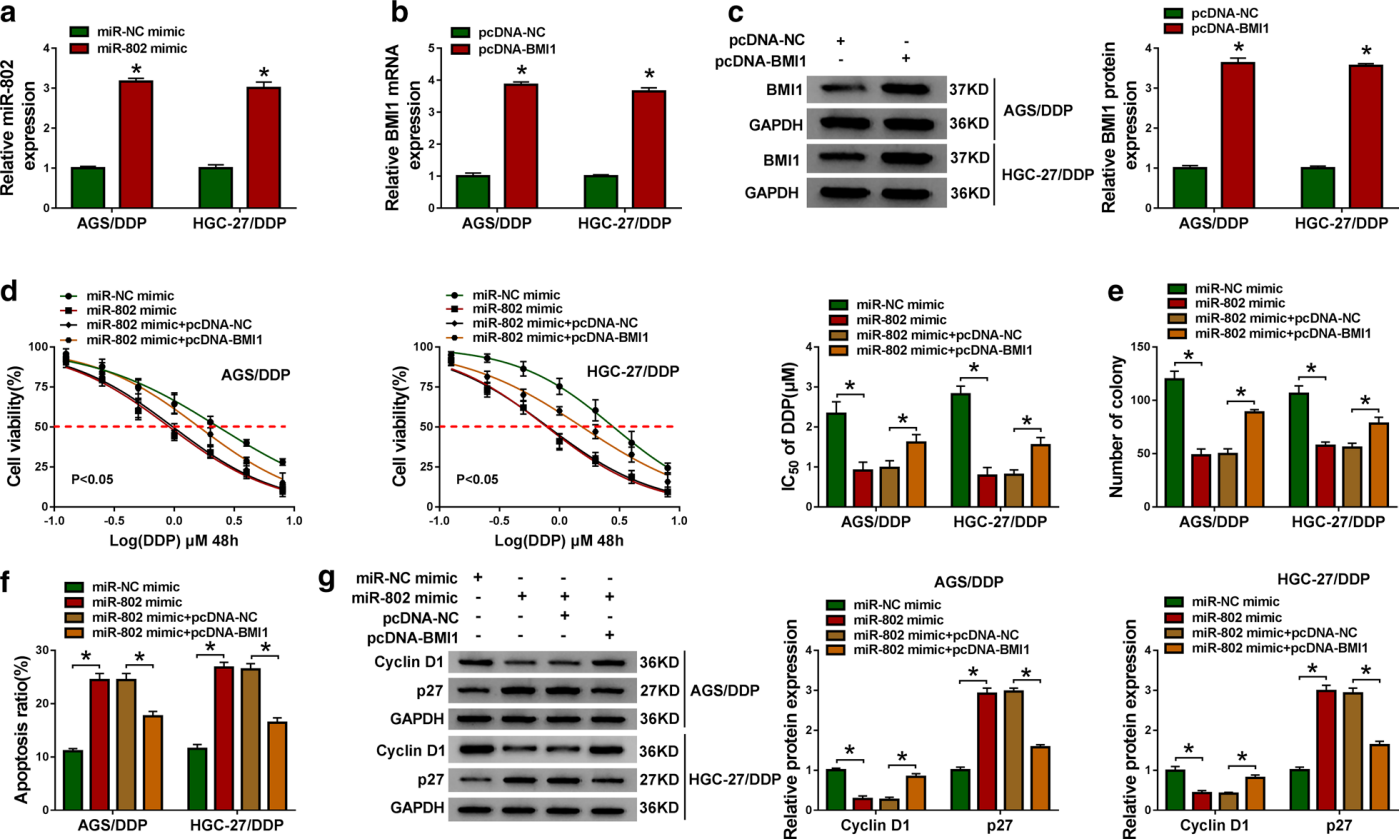
MiR-802 alleviates DDP resistance in GC cells by targeting BMI1. **a** qRT-PCR analysis of miR-802 in AGS/DDP and HGC-27/DDP cells transfected with miR-802 mimic or miR-NC mimic. **b**, **c** Levels detection of the mRNA and protein of BMI1 in AGS/DDP and HGC-27/DDP cells transfected with pcDNA-BMI1 or pcDNA-NC. AGS/DDP and HGC-27/DDP cells were transfected with miR-802 mimic, miR-NC mimic, miR-802 mimic + pcDNA-NC, or miR-802 mimic + pcDNA-BMI1. **d** CCK-8 assay of the viability and IC_50_ value of AGS/DDP and HGC-27/DDP cells after exposure to a series dose of DDP (0.125, 0.25, 0.5, 1, 2, 4, or 8 µM). **e** Colony formation assay of the colony-forming ability of AGS/DDP and HGC-27/DDP cells under 1 µM DDP treatment. **f** Flow cytometry of the apoptosis of AGS/DDP and HGC-27/DDP cells under 1 µM DDP treatment. **g** Western blot analysis of Cyclin D1 and p27 levels in AGS/DDP and HGC-27/DDP cells. n = 3, *P < 0.05

We then evaluated whether miR-802/BMI1 axis regulated DDP resistance in GC cells. First, it was proved that miR-802 mimic or pcDNA-BMI1 transfection significantly elevated the expression level of miR-802 or BMI1 in AGS/DDP and HGC-27/DDP cells compared with their counterparts, respectively (Fig. 6a–c). After that, rescue assay was conducted and results indicated cell proliferation capacity and IC_50_ value of DDP in AGS/DDP and HGC-27/DDP cells were all markedly decreased by miR-802 overexpression, while introduction with BMI1 abolished this effect (Fig. 6d, f). Furthermore, flow cytometry analysis displayed DDP-induced apoptosis was strongly increased in miR-802 mimic-transfected AGS/DDP and HGC-27/DDP cells, which was strikingly reversed by up-regulation of BMI1 (Fig. 6f). The data from western blot analysis suggested that miR-802 mimic-induced down-regulation of CyclinD1 expression and up-regulation of p27 expression was attenuated after overexpression of BMI1 in AGS/DDP and HGC-27/DDP cells (Fig. 6g). Besides that, the effects of BMI1 on AGS/DDP and HGC-27/DDP cell phenotype changes were also investigated. After elevating the expression of BMI1 in AGS/DDP and HGC-27/DDP cells using pcDNA-BMI1 overexpression vector (pcDNA-BMI1) (Additional file 4: Fig. S3A), we found BMI1 up-regulation promoted the proliferation capacity of DDP-resistant cells (Additional file 4: Fig. S3B), increased the IC_50_ value of AGS/DDP and HGC-27/DDP cells to DDP (Additional file 4: Fig. S3C), and suppressed apoptosis in AGS/DDP and HGC-27/DDP cells (Additional file 4: Fig. S3D). Collectively, these results demonstrated that miR-802 increased the sensitivity of GC cells to DDP via regulating BMI1.

### CircDONSON positively regulates BMI1 through sponging miR-802

**Fig. 7:**
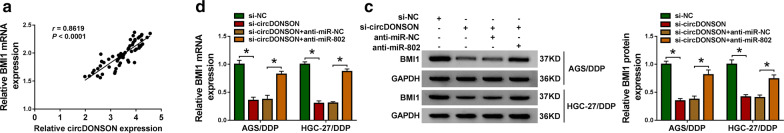
CircDONSON positively regulates BMI1 through sponging miR-802. **a** Correlation analysis between circDONSON and BMI1 expression in GC tissues. **b**, **c** Levels measurement of BMI1 mRNA and protein in AGS/DDP and HGC-27/DDP cells transfected with si-NC, si-circDONSON, si-circDONSON + anti-miR-NC, or si-circDONSON + anti-miR-802 using qRT-PCR and western blot. n = 3, *P < 0.05

Considering that miR-802 targeted BMI1, and circDONSON was a sponge of miR-802, whether circDONSON regulated BMI1 through competition for miR-802 binding was determined. First, a positive correlation between circDONSON and BMI1 level in GC tissues was observed (Fig. 7a). Next, as the exhibition of qRT-PCR and western blot analysis, we found si-circDONSON-triggered reduction of BMI1 level was significantly rescued by miR-802 inhibition in AGS/DDP and HGC-27/DDP cells (Fig. 7b, c). Altogether, circDONSON could indirectly regulate BMI1 expression via miR-802.

### CircDONSON knockdown enhances the cytotoxicity of DDP in GC in vivo

**Fig. 8:**
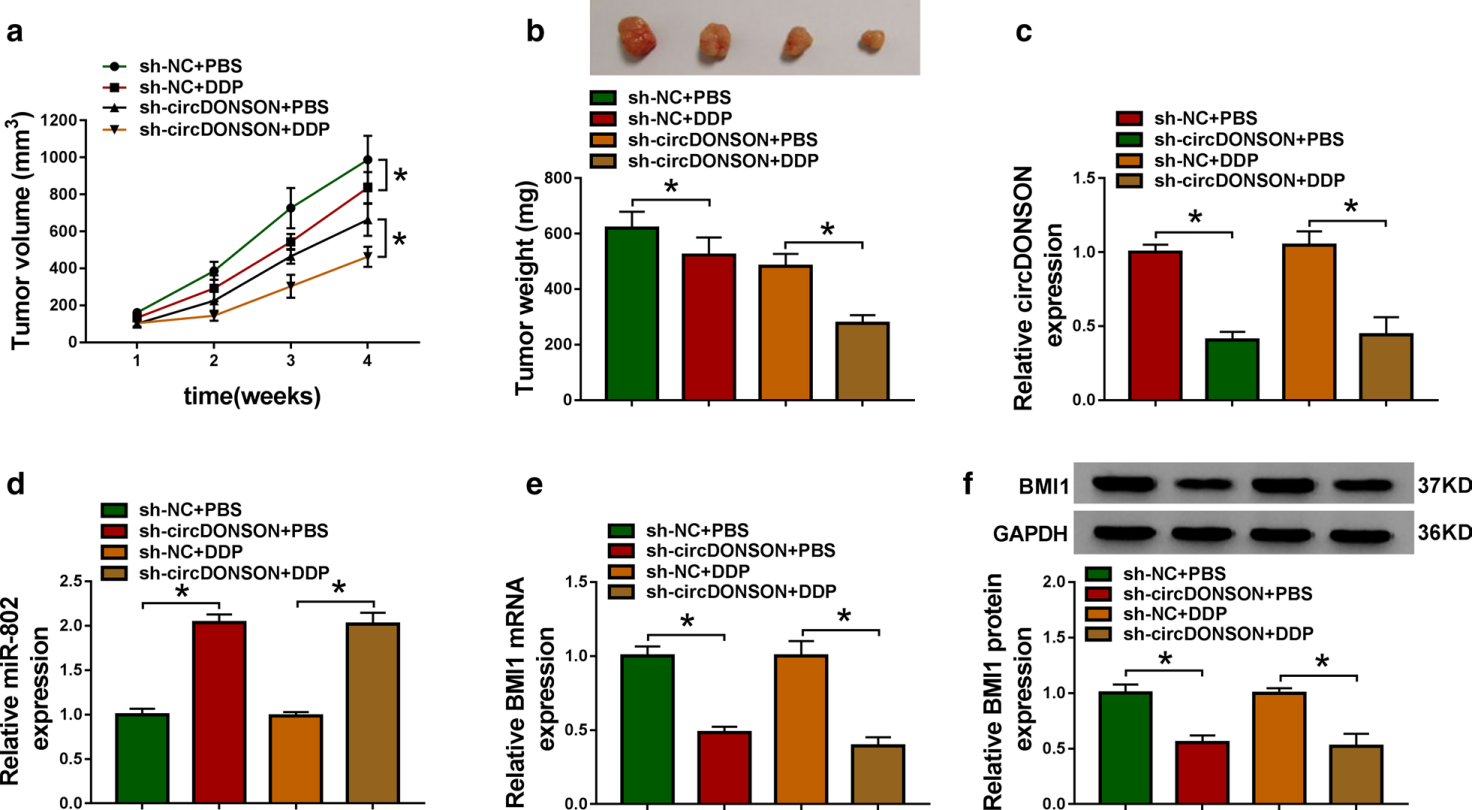
CircDONSON knockdown enhances the cytotoxicity of DDP in GC in vivo. **a** Tumor volumes were examined every week for 28 days. **b** The average weights of dissected tumors were calculated. **c**–**e** qRT-PCR analysis of circDONSON, miR-802 and BMI1 mRNA levels in dissected tumors. **f** Western blot analysis of BMI1 protein expression in dissected tumors. n = 3, *P < 0.05

The underlying effects and mechanisms of circDONSON in restoring the sensitivity of GC DDP-resistant cells to DDP in vivo were further verified. As presented in Fig. 8a and b, DDP treatment dramatically reduced tumor volume and weight compared with the control group, more importantly, a more distinct inhibition on tumor growth was observed by simultaneous circDONSON down-regulation combined with DDP treatment. Additionally, we discovered that the expression levels of circDONSON (Fig. 8c) and BMI1 (Fig. 8e, f) were decreased, while miR-802 expression was increased (Fig. 8d) in tumor masses derived from circDONSON-decrease HGC-27/DDP cells with or without DDP treatment. Overall, we concluded that knockdown of circDONSON enhanced DDP sensitivity in GC in vivo by regulating miR-802/BMI1 axis.

## Discussion

Drug resistance has been recognized as one of the difficulties for clinical tumor chemotherapy. DDP is often adopted as a first-line drug for the chemotherapy of gastric cancer patients [12], which can suppress cell vitality and kill tumor cells primarily through inducing apoptosis [13]. However, studies have shown the occurrence of DDP resistance in patients with gastric cancer, which leads to chemotherapy failure and poor prognosis [14]. Recently, several researches have demonstrated the emerging significance of circRNAs in DDP resistance in gastric cancer. For example, Huang et al. revealed that circAKT3 was higher in DDP-resistant GC tissues, and enhanced DDP resistance in GC through up-regulating PIK3R1 via miR-198 inhibition [15]. CircFN1 was found to accelerate viability and suppressed apoptosis in GC cells exposed to DDP in vitro and in vivo by binding to miR-182-5p, thus enhancing GC cell DDP resistance [16]. However, large-scale investigations of circRNA function in DDP-resistant GC cells were not yet reported.

In our present study, we focused on exploring the role of circDONSON in DDP resistance in GC cells due to its involvement in cell tumorigenesis [11]. Our results found a significant elevation of circDONSON expression in DDP-resistant GC tissues and cells, then knockdown of circDONSON restored the sensitivity of DDP-resistant cells to DDP by inhibiting cell viability and promoting cell apoptosis in vitro. Besides that circDONSON knockdown also enhanced the cytotoxicity of DDP in GC in vivo. Thus, silencing circDONSON sensitized GC cells to DDP.

Previous studies have documented that circRNAs can function as “sponges” to compete for specific miRNAs binding to enhance miRNA-mediated target genes suppression [17, 18]. Hence, we used bioinformatics analysis to search for the potential target miRNAs of circDONSON, and confirmed that miR-802 was a target of circDONSON. MiR-802 is considered as a tumor suppressor in many human cancers [19, 20]. In gastric cancer, it was found that miR-802 was down-regulated, and up-regulation of miR-802 inhibited cell proliferation, migration and invasion in GC [21, 22]. Thus, miR-802 also performs anticancer function in the development of GC. However, the role of miR-802 in DDP resistance in GC remains unclear. In this study, miR-802 was decreased in DDP-resistant GC tissues and cells, and restoration of miR-802 inhibited DDP resistance in GC cells. Importantly, silencing miR-802 reversed the inhibitory function of circDONSON-decrease in DDP-resistance in GC cells. Taken together circDONSON knockdown sensitized GC cells to DDP by targeting miR-802.

BMI1 protein is a ring finger protein encoded by BMI1 Gene, and is major component of the polycomb group complex 1 (PRC1). BMI1 is an oncogene and can regulate the proliferating, apoptotic, and invasive abilities of cancer cells, which aberrant expression is linked with the carcinogenesis and chemoresistance of numerous cancers [23, 24]. Recently, emerging evidence has found that BMI1 involved in the progression of GC. For instance, Fang et al. showed BMI1 was elevated in GC, and suppressed tumor cell apoptosis through interaction with CASC9 [25]. Liu et al. reported that knockdown of BMI1 induced apoptosis of GC cells via decreasing Bcl-2 expression and increasing caspase 3 expression [26]. Additionally, it was also demonstrated that silencing BMI1 could alleviate DDP resistance in many cancers, such as hepatocellular carcinoma [27] and squamous cell carcinoma in the head and neck [28]. Thus, BMI1 might be a useful target for overcoming DDP resistance in GC. In this study, BMI1 was found to be a target of miR-802, BMI1 was up-regulated in DDP-resistant GC tissues and cells, and overexpression of BMI1 enhanced DDP resistance and reversed the action of miR-802 on DDP resistance in GC cells. Moreover, we also confirmed circDONSON positively regulated BMI1 expression by sponging miR-802 in GC cells in vivo and in vitro.

## Conclusion

In conclusion, our findings demonstrated that circDONSON promoted cisplatin resistance in gastric cancer cells by regulating miR-802/BMI1 axis, highlighting a potential therapeutic target to overcome chemoresistance in gastric cancer patients.

## Supplementary information

**Additional file 1.** The percentages and quantification for all 4 quadrants of apoptosis.

**Additional file 2. Fig. S1** Effects of circDONSON knockdown on biochemical markers for apoptosis in vitro. Western blot analysis of Caspase-3 Cleavage and Caspase-9 Cleavage protein expression in AGS/DDP and HGC-27/DDP cells were transfected with si-NC or si-circDONSON.

**Additional file 3. Fig. S2** CircDONSON knockdown inhibits DDP resistance of GC cells in vitro. AGS/DDP and HGC-27/DDP cells were transfected with si-NC or si-circDONSON-2. After transfection, (A) qRT-PCR analysis of circDONSON expression in AGS/DDP and HGC-27/DDP cells; (B) CCK-8 assay of the viability and IC_50_ value of AGS/DDP and HGC-27/DDP cells after exposure to a series dose of DDP (0.125, 0.25, 0.5, 1, 2, 4, or 8 µM); (C) flow cytometry of the apoptosis of AGS/DDP and HGC-27/DDP cells under 1 µM DDP treatment; (D, E) western blot analysis of Caspase-3 Cleavage, Caspase-9 Cleavage, Cyclin D1 and p27 levels in AGS/DDP and HGC-27/DDP cells. n = 3, *P < 0.05.

**Additional file 4. Fig. S3** BMI1 overexpression inhibits DDP resistance of GC cells in vitro. (A) AGS/DDP and HGC-27/DDP cells were transfected with pcDNA-NC or pcDNA-BMI1. After transfection, (A) western blot analysis of BMI1 expression in AGS/DDP and HGC-27/DDP cells; (B, C) CCK-8 assay of the viability and IC_50_ value of AGS/DDP and HGC-27/DDP cells after exposure to a series dose of DDP (0.125, 0.25, 0.5, 1, 2, 4, or 8 µM); (D) flow cytometry of the apoptosis of AGS/DDP and HGC-27/DDP cells under 1 µM DDP treatment.

## Data Availability

The datasets used and/or analysed during the current study are available from the corresponding author on reasonable request.

## Abbreviations

circDONSON: Circular RNAs downstream neighbor of SON
GC: Gastric cancer
BMI1: B lymphoma Mo-MLV insertion region 1
FBS: Fetal bovine serum
GAPDH: Glyceraldehyde-3-phosphate dehydrogenase
PRC1: Polycomb group complex 1

## References

[1] Bray F, Ferlay J, Soerjomataram I, Siegel RL, Torre LA, Jemal A (2018). Global cancer statistics 2018: GLOBOCAN estimates of incidence and mortality worldwide for 36 cancers in 185 countries. Cancer J Clin..

[2] Orditura M, Galizia G, Sforza V, Gambardella V, Fabozzi A, Laterza MM, Andreozzi F, Ventriglia J, Savastano B, Mabilia A (2014). Treatment of gastric cancer. World J Gastroenterol.

[3] Chen Y, Lian G, Ou G, Yang K, Chen J, Li H, Chen S, Li J, Zeng L, Huang K (2016). Inverse association between Bmi-1 and RKIP affecting clinical outcome of gastric cancer and revealing the potential molecular mechanisms underlying tumor metastasis and chemotherapy resistance. Gastric Cancer.

[4] Pasini F, Fraccon AP, De Manzoni G (2011). The role of chemotherapy in metastatic gastric cancer. Anticancer Res.

[5] Zhu Q, Li Z, Lv C, Wang W (2019). MiR-187 influences cisplatin-resistance of gastric cancer cells through regulating the TGF-β/Smad signaling pathway. Eur Rev Med Pharmacol Sci..

[6] Ebbesen KK, Kjems J, Hansen TB (2016). Circular RNAs: identification, biogenesis and function. Biochimica et Biophysica Acta.

[7] Panda AC, Abdelmohsen K, Gorospe M (2017). SASP regulation by noncoding RNA. Mech Ageing Dev.

[8] Hombach S, Kretz M (2016). Non-coding RNAs: classification, biology and functioning Non-coding RNAs in Colorectal Cancer.

[9] Kun-Peng Z, Xiao-Long M, Chun-Lin Z (2018). Overexpressed circPVT1, a potential new circular RNA biomarker, contributes to doxorubicin and cisplatin resistance of osteosarcoma cells by regulating ABCB1. Int J Biol Sci..

[10] Liu F, Zhang J, Qin L, Yang Z, Xiong J, Zhang Y, Li R, Li S, Wang H, Yu B (2018). Circular RNA EIF6 (Hsa_circ_0060060) sponges miR-144-3p to promote the cisplatin-resistance of human thyroid carcinoma cells by autophagy regulation. Aging.

[11] Ding L, Zhao Y, Dang S, Wang Y, Li X, Yu X, Li Z, Wei J, Liu M, Li G (2019). Circular RNA circ-DONSON facilitates gastric cancer growth and invasion via NURF complex dependent activation of transcription factor SOX4. Mol Cancer..

[12] Cheng J, Cai M, Shuai X, Gao J, Wang G, Tao K (2019). First-line systemic therapy for advanced gastric cancer: a systematic review and network meta-analysis. Ther Adv Med Oncol..

[13] Goodspeed A, Jean A, Costello JC (2019). A whole-genome CRISPR screen identifies a role of MSH2 in cisplatin-mediated cell death in muscle-invasive bladder cancer. Eur Urol.

[14] Lei Y, Tang L, Hu J, Wang S, Liu Y, Yang M, Zhang J, Tang B (2020). Inhibition of MGMT-mediated autophagy suppression decreases cisplatin chemosensitivity in gastric cancer. Biomed Pharmacother.

[15] Huang X, Li Z, Zhang Q, Wang W, Li B, Wang L, Xu Z, Zeng A, Zhang X, Zhang X (2019). Circular RNA AKT3 upregulates PIK3R1 to enhance cisplatin resistance in gastric cancer via miR-198 suppression. Mol Cancer..

[16] Huang X-X, Zhang Q, Hu H, Jin Y, Zeng A-L, Xia Y-B, Xu L (2020). A novel circular RNA circFN1 enhances cisplatin resistance in gastric cancer via sponging miR-182-5p. J Cell Biochem.

[17] Su H, Tao T, Yang Z, Kang X, Zhang X, Kang D, Wu S, Li C (2019). Circular RNA cTFRC acts as the sponge of MicroRNA-107 to promote bladder carcinoma progression. Mol Cancer..

[18] Hall IF, Climent M, Quintavalle M, Farina FM, Schorn T, Zani S, Carullo P, Kunderfranco P, Civilini E, Condorelli G (2019). Circ_Lrp6, a circular RNA enriched in vascular smooth muscle cells, acts as a sponge regulating miRNA-145 function. Circ Res.

[19] Yang B, Sun L, Liang L (2019). MiRNA-802 suppresses proliferation and migration of epithelial ovarian cancer cells by targeting YWHAZ. J Ovar Res..

[20] Huang W, Shi Y, Han B, Wang Q, Zhang B, Qi C, Liu F (2020). miR-802 inhibits the proliferation, invasion, and epithelial-mesenchymal transition of glioblastoma multiforme cells by directly targeting SIX4. Cell Biochem Funct.

[21] Ma Y, Liu Y, Pu Y-S, Cui M-L, Mao Z-J, Li Z-Z, He L, Wu M, Wang J-H (2020). LncRNA IGFL2-AS1 functions as a ceRNA in regulating ARPP19 through competitive binding to miR-802 in gastric cancer. Mol Carcinog.

[22] Zhang XY, Mu JH, Liu LY, Zhang HZ (2017). Upregulation of miR-802 suppresses gastric cancer oncogenicity via targeting RAB23 expression. Eur Rev Med Pharmacol Sci..

[23] Kim M, Lee S, Park WH, Suh DH, Kim K, Kim YB, No JH (2018). Silencing Bmi1 expression suppresses cancer stemness and enhances chemosensitivity in endometrial cancer cells. Biomed Pharmacother.

[24] Ojo D, Lin X, Wu Y, Cockburn J, Bane A, Tang D (2018). Polycomb complex protein BMI1 confers resistance to tamoxifen in estrogen receptor positive breast cancer. Cancer Lett.

[25] Fang J, Chen W, Meng X-L (2019). LncRNA CASC9 suppressed the apoptosis of gastric cancer cells through regulating BMI1. Pathol Oncol Res..

[26] Liu P, Zhang M, Niu Q, Zhang F, Yang Y, Jiang X (2018). Knockdown of long non-coding RNA ANRIL inhibits tumorigenesis in human gastric cancer cells via microRNA-99a-mediated down-regulation of BMI1. Braz J Med Biol Res.

[27] Yang T, Chen Y, Zhao P, Xue H, You J, Li B, Liu Y, He C, Zhang X, Fan L (2018). Enhancing the therapeutic effect via elimination of hepatocellular carcinoma stem cells using Bmi1 siRNA delivered by cationic cisplatin nanocapsules. Nanomedicine..

[28] Chen D, Wu M, Li Y, Chang I, Yuan Q, Ekimyan-Salvo M, Deng P, Yu B, Yu Y, Dong J (2017). Targeting BMI1 cancer stem cells overcomes chemoresistance and inhibits metastases in squamous cell carcinoma. Cell Stem Cell.

