# Supplementary material for: CircRNA DONSON contributes to cisplatin resistance in gastric cancer cells by regulating miR-802/BMI1 axis
Source: Cancer Cell Int. 2020 Jun 22;20:261. doi: 10.1186/s12935-020-01358-w (PMC7310092; doi:10.1186/s12935-020-01358-w)
Supplement: Supplementary file 1 — Additional file 1. The percentages and quantification for all 4 quadrants of apoptosis. [file 12935_2020_1358_MOESM1_ESM.doc]

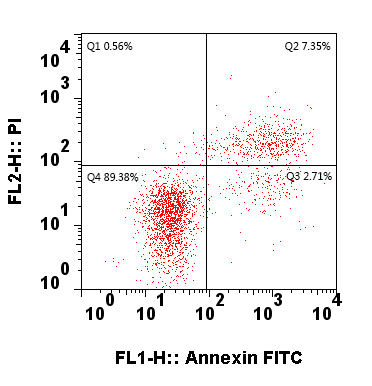


| Popilation | Events | %Parent |
| --- | --- | --- |
| All Events | 100000 | 100.00% |
| Q1 | 560 | 0.56% |
| Q2 | 7350 | 7.35% |
| Q3 | 2710 | 2.71% |
| Q4 | 89380 | 89.38% |


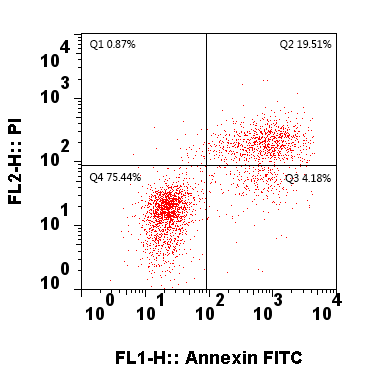


| Popilation | Events | %Parent |
| --- | --- | --- |
| All Events | 100000 | 100.00% |
| Q1 | 870 | 0.87% |
| Q2 | 19510 | 19.51% |
| Q3 | 4180 | 4.18% |
| Q4 | 75440 | 75.44% |


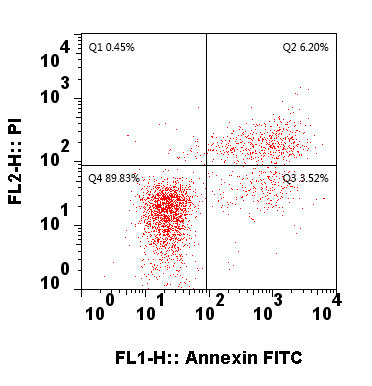


| Popilation | Events | %Parent |
| --- | --- | --- |
| All Events | 100000 | 100.00% |
| Q1 | 450 | 0.45% |
| Q2 | 620 | 6.20% |
| Q3 | 3520 | 3.52% |
| Q4 | 89830 | 89.83% |


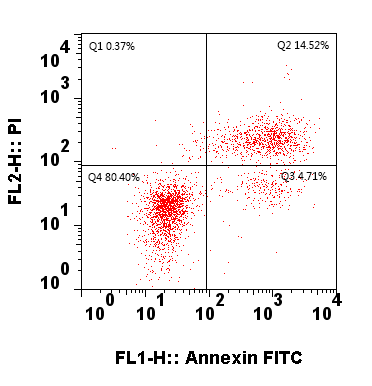


| Popilation | Events | %Parent |
| --- | --- | --- |
| All Events | 100000 | 100.00% |
| Q1 | 370 | 0.37% |
| Q2 | 14520 | 14.52% |
| Q3 | 4710 | 4.71% |
| Q4 | 80400 | 80.40% |
