# Supplementary figures and images for: CircRNA DONSON contributes to cisplatin resistance in gastric cancer cells by regulating miR-802/BMI1 axis
Source: Cancer Cell Int. 2020 Jun 22;20:261. doi: 10.1186/s12935-020-01358-w (PMC7310092; doi:10.1186/s12935-020-01358-w)

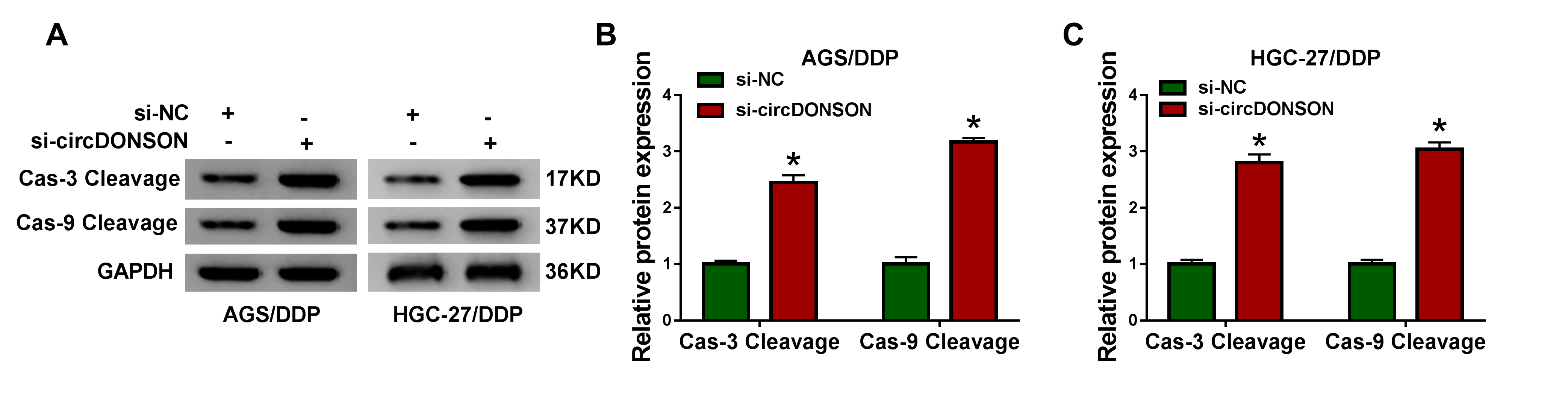

Supplement: Supplementary file 2 — Additional file 2. Fig. S1 Effects of circDONSON knockdown on biochemical markers for apoptosis in vitro. Western blot analysis of Caspase-3 Cleavage and Caspase-9 Cleavage protein expression in AGS/DDP and HGC-27/DDP cells were transfected with si-NC or si-circDONSON. [file 12935_2020_1358_MOESM2_ESM.tif]

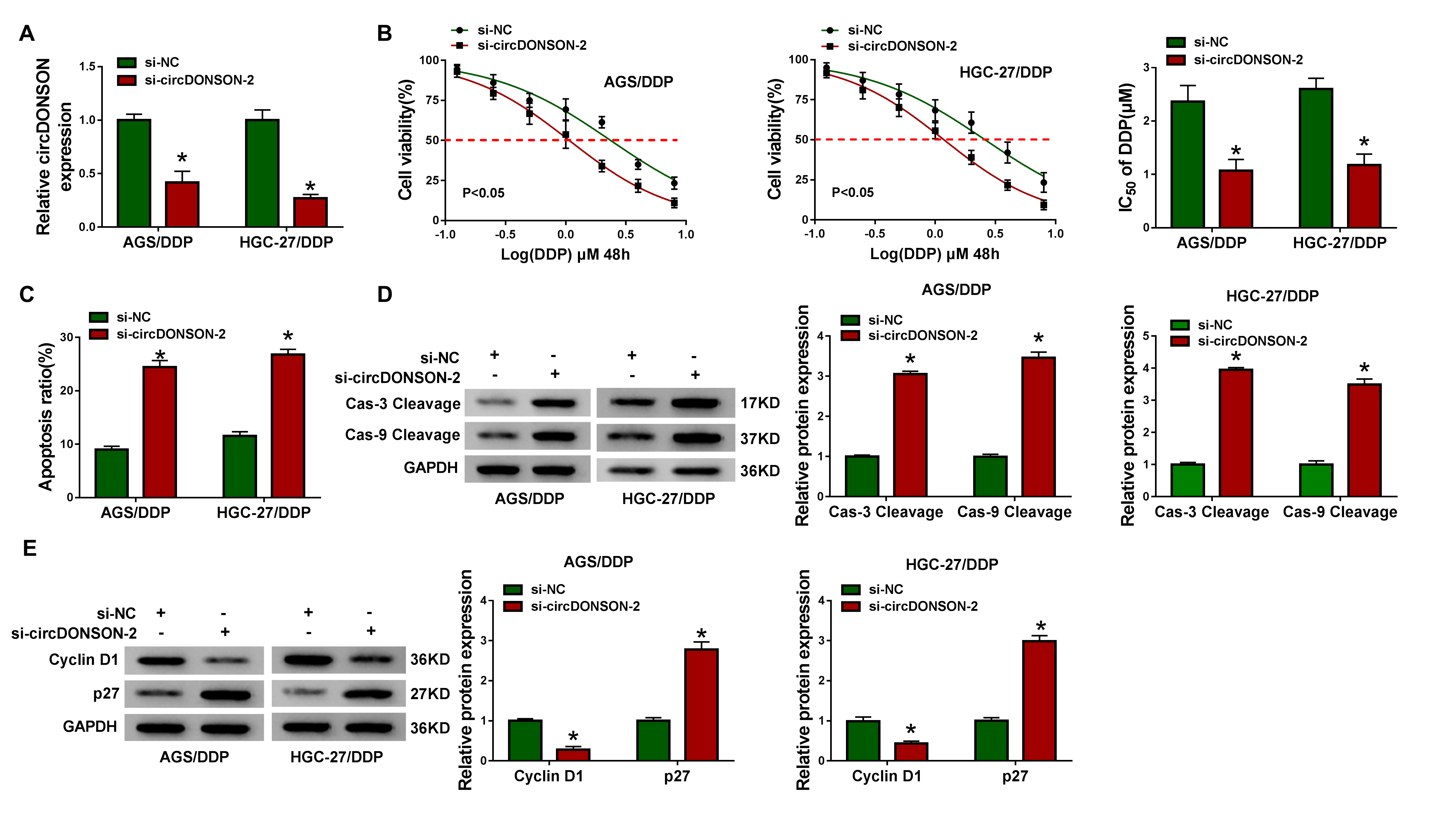

Supplement: Supplementary file 3 — Additional file 3. Fig. S2 CircDONSON knockdown inhibits DDP resistance of GC cells in vitro. AGS/DDP and HGC-27/DDP cells were transfected with si-NC or si-circDONSON-2. After transfection, (A) qRT-PCR analysis of circDONSON expression in AGS/DDP and HGC-27/DDP cells; (B) CCK-8 assay of the viability and IC50 value of AGS/DDP and HGC-27/DDP cells after exposure to a series dose of DDP (0.125, 0.25, 0.5, 1, 2, 4, or 8 µM); (C) flow cytometry of the apoptosis of AGS/DDP and HGC-27/DDP cells under 1 µM DDP treatment; (D, E) western blot analysis of Caspase-3 Cleavage, Caspase-9 Cleavage, Cyclin D1 and p27 levels in AGS/DDP and HGC-27/DDP cells. n = 3, *P < 0.05. [file 12935_2020_1358_MOESM3_ESM.tif]

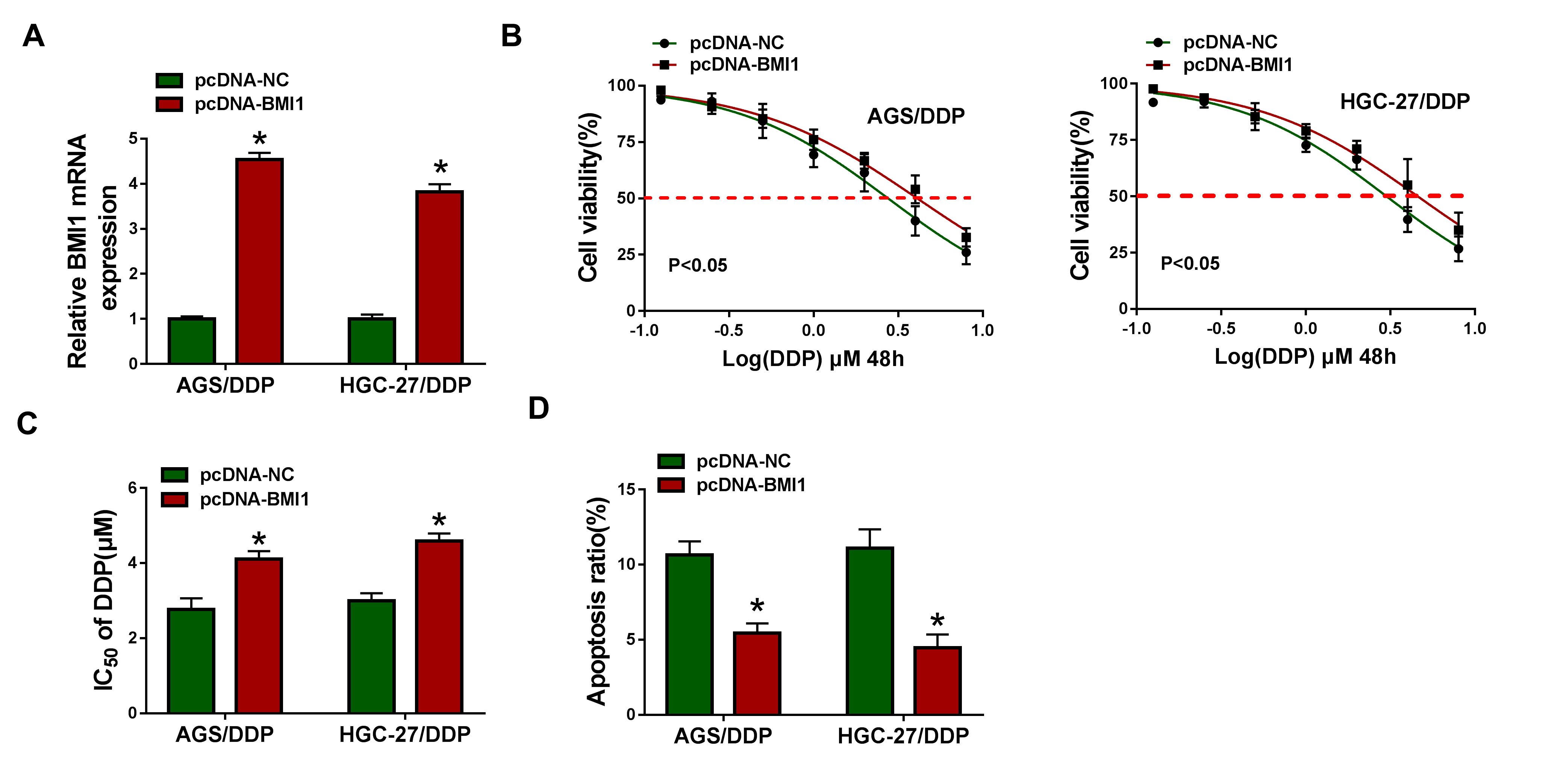

Supplement: Supplementary file 4 — Additional file 4. Fig. S3 BMI1 overexpression inhibits DDP resistance of GC cells in vitro. (A) AGS/DDP and HGC-27/DDP cells were transfected with pcDNA-NC or pcDNA-BMI1. After transfection, (A) western blot analysis of BMI1 expression in AGS/DDP and HGC-27/DDP cells; (B, C) CCK-8 assay of the viability and IC50 value of AGS/DDP and HGC-27/DDP cells after exposure to a series dose of DDP (0.125, 0.25, 0.5, 1, 2, 4, or 8 µM); (D) flow cytometry of the apoptosis of AGS/DDP and HGC-27/DDP cells under 1 µM DDP treatment. [file 12935_2020_1358_MOESM4_ESM.tif]
